# Sharing extended summary data from contemporary genetics studies is unlikely to threaten subject privacy

**DOI:** 10.1371/journal.pone.0179504

**Published:** 2017-06-29

**Authors:** Silviu-Alin Bacanu

**Affiliations:** Department of Psychiatry, Virginia Institute for Psychiatric and Behavioral Genetics, Virginia Commonwealth University, Richmond, Virginia, United States of America; University of North Carolina at Chapel Hill, UNITED STATES

## Abstract

**Background:**

Starting from a forensic problem, Homer et al. showed that it was possible to detect if an individual contributes only 0.5% of the DNA in a pool. The finding was extended to prove the possibility of detecting whether a subject participated in a small homogeneous GWAS. We denote this as the detection of a **s**ubject **b**elonging to a **c**ertain **c**ohort (SBCC). Subsequently, Visscher and Hill showed that the power to detect SBCC signal for an ethnically homogeneous cohort depends roughly on the ratio of the number of independent markers and total sample size. However, it is not clear if the same holds for more ethnically diverse cohorts. Later, Masca et al. propose running as SBCC test a regression of departure from assumed population frequency of i) subject genotype on ii) cohort of interest frequency. They use simulations to show that the approach has better SBCC detection power than the original Homer method but is impeded by population stratification.

**Approach:**

To investigate the possibility of SBCC detection in multi-ethnic cohorts, we generalize the Masca et al. approach by theoretically deriving the correlation between a subject genotype and the cohort reference allele frequencies (RAFs) for stratified cohorts. Based on the derived formula, we theoretically show that, due to background stratification noise, SBCC detection is unlikely even for mildly stratified cohorts of size greater than around a thousand subjects. Thus, for the vast majority of contemporary cohorts, the fear of compromising privacy via SBCC detection is unfounded.

## Introduction

Spurred by stricter NIMH requirement for sharing data, in the beginning of Genome Wide Association Studies (GWASs) era most researchers published in a timely manner summary statistics from studies, e.g. Z-scores, odds ratios (OR) and, even reference allele frequency (RAF) by case status. However, this free sharing did not last long before privacy concerns were raised. First, Homer et al.[1], starting from a forensic problem, showed that it was possible to detect if an individual contributes only 0.5% of the DNA in a pool. In the same paper, the authors extended the findings to show that you can detect if a subject participated in a small (N≈1,500) homogeneous GWAS by using only summary statistics and RAFs. We denote this the detection of a **s**ubject **b**elonging to a **c**ertain **c**ohort (SBCC).

Subsequently, Visscher and Hill [2] used a likelihood ratio (LR) approach to show that the power to detect SBCC signal for an ethnically homogeneous cohort depends roughly on the ratio of the number of independent markers and total sample size. Unfortunately, even though Visscher and Hill implied that at larger sample sizes the power of detecting whether a subject is the member of a cohort is much smaller, this finding was not enough to avoid a chilling effect on the free sharing of summary data.

By using a Bayesian approach Clayton [3] investigated the conditions needed for SBCC detection for a homogeneous cohort. He computes Bayes factors for subject belonging to case and control group and derives their upper limit as a function of allele frequency. He also touches on the lack of good reference data making SBCC even harder. In the end, Clayton concludes that that “scenarios in which an individual might be identified in this manner are somewhat improbable—particularly when so many SNPs would be needed that linkage disequilibrium could not be ignored (so that any potential invader of privacy would also require access to an individual-level data set from which to estimate the linkage disequilibrium structure)”.

Later, Masca et al. [4] propose as SBCC statistic an empirical regression test of departure from assumed population frequency of i) subject genotype on ii) cohort of interest frequency. They use simulations to show that i) their approach is more powerful than Homer et al., ii) population stratification impedes SBCC detection and ii) SBCC detection is possible only at smaller sizes.

In this paper we attempt to answer the question whether, from an SBCC perspective, not sharing data is scientifically defensible for present day GWAS studies. To answer it we theoretically extend Masca et al SBCC approach, ii) update it for stratified cohorts and ii) use the approach for SBCC signal testing. As a measure of SBCC signal strength we propose the **c**orrelation between a subject **g**enotype and the cohort **R**AFs (CGR). We show that for unstratified cohorts, CGR is equivalent to Visscher and Hill LR, which suggest our approach is locally uniform most powerful (UMP) test under modest stratification. Based on the functional form of CGR statistic we argue that, for the vast majority of contemporary cohorts, stopping the free sharing of data due to SBCC concerns is not scientifically justified.

## Methods

Given that the information relating to SBCC for certain disorders is likely to be much more detrimental than him/her belonging to the cohort of a quantitative trait, in this paper the focus in on case control cohorts. Due to subjects’ contribution to i) the Z-scores being adjusted for unknown ancestry components and ii) RAFs incorporating solely unadjusted subjects’ contribution, we argue that RAFs are likely to provide much more information on whether a subject belongs to a cohort. Consequently, this paper will treat only the privacy concerns relating to the worst-case scenario of sharing case RAFs.

### Correlation between case genotype and in-cohort RAF

Assume the cohort under investigation consists of *n* cases and *n*′ controls for a certain disorder. Further assume that the cohort samples *m* subpopulations, with the *i*-th subpopulation having *n*_*i*_ cases and *n*′_*i*_ controls. Under stratification, an important index for population divergence is Wright’s fixation index *F*_*st*_, which is the quotient of the variance in subpopulation frequencies and the variance of the allele in cohort (*1*). *F*_*st*_ was also shown to be the apparent correlation of alleles in the same subpopulation (*1*). (Alleles from different subpopulations are uncorrelated.) Let *F*_*i*_ denote the correlation of the alleles in the *i*-th subpopulation.

Before proceeding to deduce the correlation between case genotype and in-cohort RAF, i.e. CGR, we establish some basic relationships for variance and covariance of subjects’ genotypes. Assume that *X*_1_ and *X*_2_ are the additively coded alleles (i.e. the number of reference alleles) of an individual from the *i*-th subpopulation, then the genotype *G* = *X*_1_ + *X*_2_. Then, *Var*(*G*) = *Var*(*X*_1_ + *X*_2_) = *Var*(*X*_1_) + *Var*(*X*_2_) + 2 *Cov*(*X*_1_ + *X*_2_), i.e.

$$Var\left(G\right)=p\left(1-p\right)+p\left(1-p\right)+2p\left(1-p\right)F_{i}=2p\left(1-p\right)\left(1+F_{i}\right)$$ (1)

Let *G*_*1*_ = *X*_11_ + *X*_12_ and *G*_2_ = *X*_21_ + *X*_22_ be the bi-allelic genotype for 2 subjects from the same subpopulation (with fixation index *F*_*i*_) or two different subpopulations. Then *Cov*(*G*_1_, *G*_2_) = *Cov*(*X*_11_ + *X*_12_, *X*_21_ + *X*_22_) = *Cov*(*X*_11_, *X*_21_) + *Cov*(*X*_11_, *X*_22_) + *Cov*(*X*_12_, *X*_21_) + *Cov*(*X*_12_, *X*_22_) = 4 *Cov*(*X*_11_, *X*_21_) Eq (2). Thus,

$$Cov\left(G_{1},G_{2}\right)=\{\begin{array}{cc} 4p\left(1-p\right)F & \text{subjects from same population}\\ 0 & \text{otherwise} \end{array}$$ (2)

With these notations, assume that *G*_*i*,*j*_ (*G′*_*i*,*j*_), *i* = 1, …, *m* and $j=1,\ldots n_{i}\left(n_{i}\prime \right)$ are the additively coded genotype at the variant under investigation for the *j*-th individual in the *i-*th subpopulation in the cases (controls). For this variant, having a population RAF of *p*, let ${\hat{p}}_{A}=\frac{\sum_{i=1}^{m}\sum_{j=1}^{n_{i}}G_{i,j}}{2\;n}$ and ${\hat{p}}_{U}=\frac{\sum_{i=1}^{m}\sum_{j=1}^{n\prime_{i}}G\prime_{i,j}}{2\;n\prime }$ be the estimated allele frequency in the affected (cases) and unaffected (controls) subjects, respectively. Suppose studies publicly report RAF estimate of the form: $\hat{p}=\omega \;{\hat{p}}_{A}+\left(1-\omega \right){\hat{p}}_{U}.$ For example, from a population genetics point of view might be of interest to report $\hat{p}$ for *ω* = *K*, i.e. the population RAF estimate. [Other interesting scenarios is to report both ${\hat{p}}_{A}$ (ω = 1) and ${\hat{p}}_{U}$(*ω* = 0).]

Assuming that the study reports such $\hat{p}$ estimates for all common SNPs, for privacy considerations it is desirable to compute the expected correlation between a certain case genotype, *G*_*i′*,*j′*_, and $\hat{p}$. To this end we start by first estimating $\;Var\left(\hat{p}\right)$ and $E[\left(G_{i^{\prime },j^{\prime }}-2\;p\right)(\;\hat{p}-p)]$. Relationship Eqs (1) and (2) from above [also in Devlin et al.(*1*)], can be re-written as: $Var(G_{i,j})=Var(G\prime_{i,j})=2p\left(1-p\right)\left(1+F_{i}\right)$ and $Cov\left(G_{i,j},G_{i,j^{\prime }}\right)=Cov\left(G\prime_{i,j},G_{i,j^{\prime }}\right)=Cov\left(G_{i,j},G\prime_{i,j^{\prime }}\right)=4\;p\left(1-p\right)F_{i}$ and *Cov*(*G*_*i′*,*j*_,*G*_*i*,*j′*_) = 0 for *i*′≠ *i*.

With these relationships $Var\left(\hat{p}\right)=Var\left(\frac{\omega }{2\;n}\;\sum {}_{i=1}^{m}\sum_{j=1}^{n_{i}}G_{i,j}+\frac{\left(1-\omega \right)}{2\;n^{\prime }}\;\sum {}_{i=1}^{m}\sum {}_{j=1}^{n^{\prime }}{G^{\prime }}_{i,j}\right)$ becomes $Var\left(\hat{p}\right)=2p\left(1-p\right)\left\{\frac{\omega^{2}}{4\;n^{2}}\left[n\left(1+F_{i}\right)+2\overset{m}{\underset{i=1}{\sum }}n_{i}\left(n_{i}-1\right)\;F_{i}\right]\;+\frac{4\omega \left(1-\omega \right)}{4\;n\;n^{\prime }}\overset{m}{\underset{i=1}{\sum }}n_{i}{n^{\prime }}_{i}F_{i}+\frac{{\left(1-\omega \right)}^{2}}{4\;{n^{\prime }}^{2}}\left[n^{\prime }\left(1+F_{i}\right)+2\;\overset{m}{\underset{i=1}{\sum }}{n^{\prime }}_{i}\left({n^{\prime }}_{i}-1\right)F_{i}\right]\right\}$. Similarly, $\begin{array}{l} E[\left(G_{i^{\prime },j^{\prime }}-2\;p\right)\left(\;\hat{p}-p\right)]=E[\left(G_{i^{\prime },j^{\prime }}-2\;p\right)[\frac{\omega }{2\;n}\;\Sigma_{i=1}^{m}\Sigma_{j=1}^{ni}(G_{i,j}-2\;p)+\frac{\left(1-\omega \right)}{2\;n^{\prime }}\;\Sigma_{i=1}^{m}\Sigma_{j=1}^{{n^{\prime }}_{i}}({G^{\prime }}_{i,j}-2\;p)]\} \end{array}$ simplifies to $E\left[\left(G_{i^{\prime },j^{\prime }}-2p\right)\left(\;\hat{p}-p\right)\right]=2p\left(1-p\right)\left\{\frac{\omega }{2\;n}\left[1+F_{i^{\prime }}+2*\left(n_{i^{\prime }}-1\right)F_{i^{\prime }}\right]+2\frac{\left(1-\omega \right)}{2\;n^{\prime }}{n^{\prime }}_{i^{\prime }}F_{i^{\prime }}\right\}.$ Thus, given that *Var*(*G*_*i′*,*j′*_) = 2*p*(1 − *p*)(1 + *F*_*i*_), the correlation of interest becomes:

$$Cor\left(G_{i\prime ,j\prime }\;\hat{p}\right)=\frac{\frac{\omega }{2n}\left[1+\left(2\;n_{i^{\prime }}-1\right)F_{i^{\prime }}\right]+\;\frac{\left(1-\omega \right)n}{n\prime }n_{i}^{\prime }F_{i^{\prime }}}{\sqrt{\left(1+F_{i}\right)\left(\frac{\omega^{2}}{4n^{2}}\left[n\left(1+F_{i}\right)+2\sum_{i=1}^{m}n_{i}\left(n_{i}-1\right)F_{i}\right]\;+\frac{4\omega \left(1-\omega \right)}{4nn^{\prime }}\sum_{i=1}^{m}n_{i}{n^{\prime }}_{i}F_{i}+\frac{{\left(1-\omega \right)}^{2}}{\;4{n^{\prime }}^{2}}\left[n^{\prime }\left(1+F_{i}\right)+2\;\sum_{i=1}^{m}{n^{\prime }}_{i}\left({n^{\prime }}_{i}-1\right)F_{i}\right]\right)}}.$$

Further manipulations, reduces the correlation to:

$$Cor\left(G_{i\prime ,j\prime }\;\hat{p}\right)=\frac{1+\left(2\;n_{i^{\prime }}-1\right)F_{i^{\prime }}+2\;\frac{\left(1-\omega \right)n}{\omega n\prime }n_{i^{\prime }}F_{i^{\prime }}}{\sqrt{\left(1+F_{i}\right)\left[n\left(1+F_{i}\right)+2\sum_{i=1}^{m}n_{i}\left(n_{i}-1\right)F_{i}\right]\;+\frac{4\left(1-\omega \right)n}{\omega \;n^{\prime }}\sum_{i=1}^{m}n_{i}{n^{\prime }}_{i}F_{i}+\frac{{\left(1-\omega \right)}^{2}\;n^{2}}{\;\omega^{2}{n^{\prime }}^{2}}\left[n^{\prime }\left(1+F_{i}\right)+2\;\sum_{i=1}^{m}{n^{\prime }}_{i}\left({n^{\prime }}_{i}-1\right)F_{i}\right]}}.$$

If we assume the same *F*_*st*_ for all populations and an equal number of cases and controls in each subpopulation, i.e. *F*_*i*_ = *F* and $n_{i}=n\prime_{i}=\frac{n}{m}$, for large numbers the formula is approximated by:

$$Cor\left(G_{i\prime ,j\prime }\;\hat{p}\right)≅\frac{1+2\;\left[1+\frac{\left(1-\omega \right)}{\omega }\right]\frac{n}{m}F}{\sqrt{\left(1+F\right)\left(\left[1+\frac{{\left(1-\omega \right)}^{2}}{\omega^{2}}\right]n\;+\left[1+\frac{2\;\left(1-\omega \right)}{\omega }+{\frac{\left(1-\omega \right)}{\omega }}^{2}\right]\frac{2\;n^{2}}{m}F\right)}}$$

Thus, under stratification, the correlation between the genotype of a case (*ω* = 1, above) and the allele frequency of cases can be approximated by

$$\rho \left(F\right)=Cor\left(G_{i\prime ,j\prime }\;\hat{p}\right)=\frac{1+2\;\frac{n}{m}F}{\sqrt{\left(1+F\right)\left(n\;+\frac{2\;n^{2}}{m}F\right)}}$$ (3)

The functional form from equation form was empirically validated [see subsection 1.3 and Fig A in S1 File]. The correlation between a subject genotype and RAF can be also estimated for a subject not belonging to the cohort (subsection 1.1 in SM).

### Using correlation between case genotype and in-cohort RAF to test SBCC

*ρ*(*F*) from Eq 3 can be approximated via first order Taylor series:

$$\rho \left(F\right)=\frac{1}{\sqrt{n}}+\frac{\sqrt{n}}{\;m}\;F=\rho \left(0\right)+\frac{\sqrt{n}}{\;m}\;F$$

(for more details, see Eqs B and C in S1 File).

Because the $\frac{\sqrt{n}}{\;m}\;F$ bias might not be negligible even for moderately sized intracontinental meta-analyses, to test the true correlation due to belonging to the case cohort -$\rho \left(0\right),\frac{\sqrt{n}}{\;m}\;F$ bias needs to be subtracted. Based on the above Taylor series approximation, *ρ*(0)can be estimated by $\;\hat{\rho \left(0\right)}\;=\;≅\hat{\rho \left(F\right)}-\frac{\sqrt{n}}{m}\;\tilde{F}$, where $\tilde{F}$ is estimated using a relevant and ideal, i.e. perfectly matching ethnic distribution, panel of size $n^{\prime \prime }=\frac{n}{k}$ (*k* >> 10 for large meta-analyses). It follows that $Var\left[\hat{\rho \left(0\right)}\right]=\frac{1}{o}+\frac{\;k}{m^{2}}$ (Eq 4 in subsection 1.4 of SM), where *o* is the equivalent number of independent SNPs in genome scan. Thus the expectation of Z-score for testing *ρ*(0) = 0 (subject not in cohort) vs. *ρ*(0) > 0 (which likely yields higher power than testing the more appropriate *ρ*(0) = 0 vs $\rho \left(0\right)=\frac{1}{\sqrt{n}}$ [subject in cohort]), is

$$\mu =\frac{\frac{1}{\sqrt{n}}}{\sqrt{\frac{1}{o}+\frac{k}{m^{2}}}}=\frac{1}{\sqrt{\frac{n}{o}+\frac{kn}{m^{2}}}}$$ (4)

for subjects in the case cohort. We stress that if non-stratification is assumed (i.e. to eliminate $\frac{kn}{m^{2}}$ in relationship (4)), the equivalent *X*^2^test has the noncentrality parameter $\lambda =\mu^{2}=\frac{o}{n}$ which is similar to the one deduced by Visscher and Hill using a likelihood ratio (LR) approach when either i) not augmenting the data with a reference panel and ii) being able to use the cohort sample along with reference panel to estimate $\tilde{F}$. Given the desirable properties of LR tests [5] (Theorem 8.3.1-Neyman Pearson Lemma)) and the fact that *F* is very small in practice (e.g. *F* = 0.006 in the most divergent European populations [6]) it follows that test based on relationship (4) is UMP or close to UMP for modest stratification. Assuming (extremely) conservatively that the number of independent SNPs is *o* = ∞, instead of *o* = 50,000 as in [2], we compute the upper bound for the probability (power) of detecting a significant signal for subjects belonging to case cohort at a certain type I error, *α*, is

$$q=\Phi \;(\frac{1}{\sqrt{\frac{kn}{m^{2}}}}-\tau_{\alpha })$$

where *τ*_*α*_ = *ϕ*^−1^(1−*α*).

### Simulated scenarios used to evaluate power to detect SBCC

To give an idea about power to detect SBCC signal we present a range of scenarios inspired by existing data sets. As possible values of the parameters (present and future) we chose: panel sample size of $n^{\prime \prime }=\frac{n}{k}=\left\{1,000;\;10,000;\;30,000;\;100,\;000\right\}$, and the number of subpopulations set to $m=\text{max}\left(〚\frac{n}{n_{s}}〛,2\right)$, where 〚.〛 is the rounding to the nearest integer function and, rather conservatively, (as multiple studies target the same subpopulation) *n*_*s*_ = {700; 1,400, 2,800} is the average number of cases per study. The values for the number of cases per study is informed by the analysis of the second schizophrenia cohort from the Psychiatric Genetics Consortium (PGC) [7], which averages 700 cases per study. The assumptions regarding *n*_*s*_ are conservative because i) in many large studies (PGC included) multiple sub-studies are targeting the same subpopulation and ii) with the increase of total sample sizes of meta-analyses the sample sizes coming from each subpopulation are expected to increase.

### Practical application

We apply the method to PGC2 schizophrenia (SCZ) [7]. It discovered 108 loci by analyzing a multiethnic cohort which included slightly more than 30,000 cases. Each individual study contributed around 700cases. We assume that $\tilde{F}$ is estimated using the publicly available subpanel of Haplotype Reference Consortium [8], which contains around *n*″ = 12,000 subjects.

## Results

**Fig 1:**
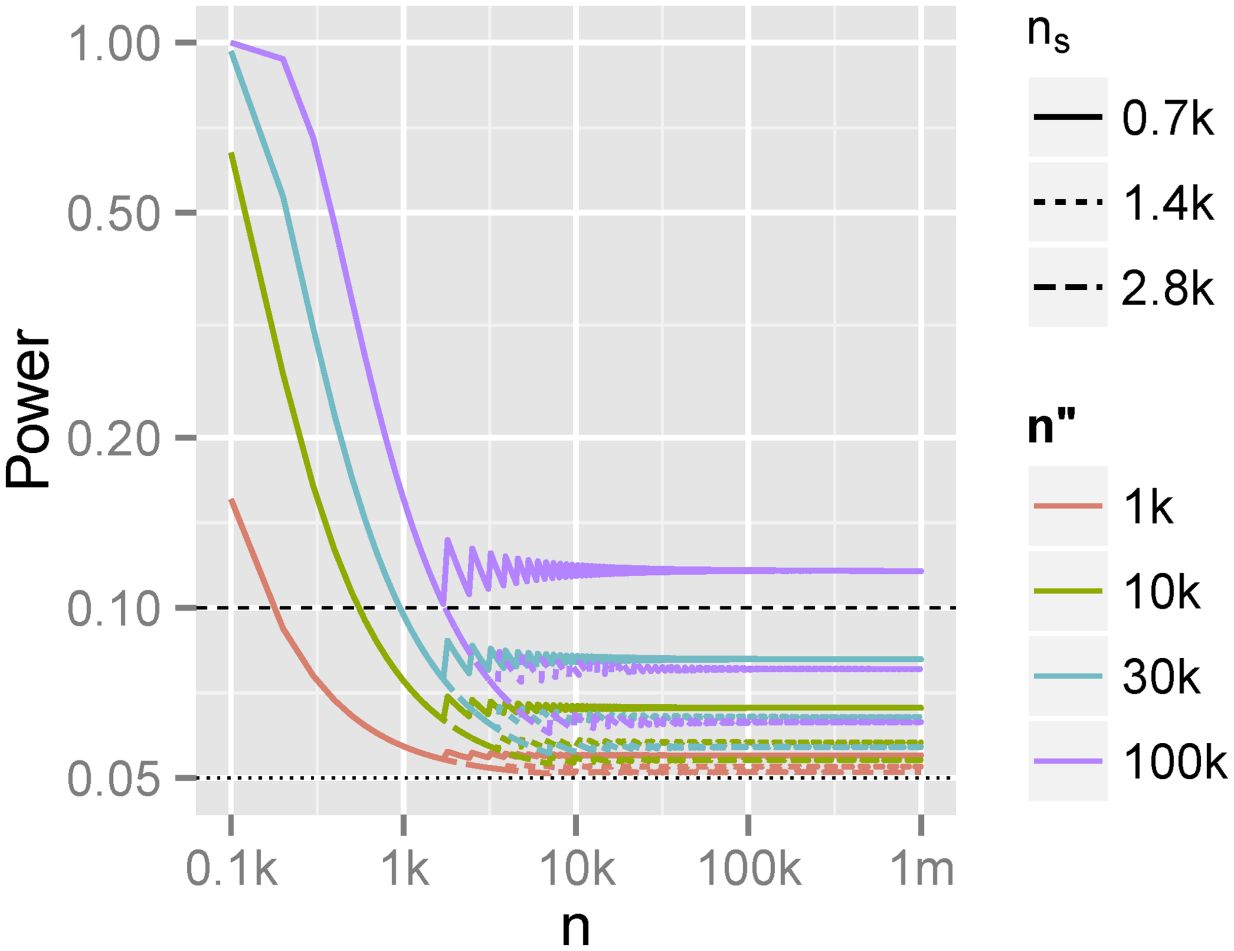
Detection power as a function of the sizes of the meta-analysis (n, for which k denotes thousands and m millions), panel (*n*″) and cases cohort in individual studies of meta-analysis (*n*_*s*_). Dotted/dashed lines correspond to detection power of 0.05 (type I error)/0.1.

With these conservative assumptions, we obtain an upper limit for the detection power, *q*, as a function of sample size, *n* (Fig 1). These calculations show that, at a type I error of 0.05, there is some modest power to detect the case belonging signal (Fig 1) only when i) (perfectly matching) panel size is extremely large *and* ii) cohort size is lower than 1,000. For more realistic parameter scenarios, the power of detection is practically negligible.

For the practical application to PGC2 SCZ, assuming 700 cases per individual study and n″ = 12,000, the power to detect SBCC signal is around 6.6% for a type I error rate of *α* = 5%. If using the smaller 1000 Genome reference phase 1 [9] (*n*″ = 1,000) and 3 [10](*n*″ = 2,504) the power decreases to 5.7% and 5.5%, respectively. However, even such near-false-positive-rate detection powers are likely overestimates due to poor panel coverage of many PGC2 SCZ subpopulations.

## Discussion

SBCC related privacy concerns do not preclude sharing summary data (even *case RAFs*) even when analyzing cohorts of rather modest stratification and size. This is due SBCC signal (for a cohort of size > ~ 1,000) being overwhelmed by the stratification background noise even when very large reference panels are available. Consequently, as far as SBCC detection is concerned, there is no scientifically valid reason why the summary data for most genetic studies, including case RAFs, should not be made publicly available. However, our work does not preclude data sharing raising privacy concerns from, currently unidentified, non-SBCC vantage points.

## Supporting information

S1 File (DOCX)
