# Supplementary material for: Sharing extended summary data from contemporary genetics studies is unlikely to threaten subject privacy
Source: PLoS One. 2017 Jun 29;12(6):e0179504. doi: 10.1371/journal.pone.0179504 (PMC5491025; doi:10.1371/journal.pone.0179504)
Supplement: S1 File — (DOCX) [file pone.0179504.s001.docx]

- 1. **Correlation between case genotype and in-cohort reference allele frequency (RAF)**

Assume the cohort under investigation consists of $n$ cases and $n'$ controls for a disorder with a prevalence $K$. Further assume that the cohort samples $m$ subpopulations, with the $i$-th subpopulation having $n_{i}$ cases and ${n'}_{i}$ controls and, with respect to the overall cohort RAF, an apparent correlation of alleles (also denoted as fixation index, $F_{st}$) in a subpopulation equal to $F_{i}$ (*1*).

With these notations, assume that $G_{i,j}$ (${G'}_{i,j}$),$i=1,\ldots,m$ and $j=1,\ldots n_{i}(n_{i}')$ are the additively coded genotype at the variant under investigation for the $j$-th individual in the $i$-th subpopulation in the cases (controls). For this variant, having a population RAF of $p$, let $\hat{p}_{A}=\frac{\sum_{i=1}^{m} \sum_{j=1}^{n_{i}} G_{i,j}}{2 n}$ and $\hat{p}_{U}=\frac{\sum_{i=1}^{m} \sum_{j=1}^{{n'}_{i}} {G'}_{i,j}}{2 n'}$ be the estimated allele frequency in the affected (cases) and unaffected (controls) subjects, respectively. Suppose studies publically report RAF estimate of the form: $\hat{p}=\omega\hat{p}_{A}+\left( 1-\omega\right)\hat{p}_{U}.$For example, from a population genetics point of view might be of interest to report $\hat{p}$ for $\omega=K$, i.e. the population RAF estimate. (Other interesting scenarios is to report both $\hat{p}_{A}$ ($\omega=1$) and $\hat{p}_{U}$ ($\omega=0$).)

Assuming that the study reports such $\hat{p}$ estimates for all common SNPs, for privacy considerations it is desirable to compute the expected correlation between a certain case genotype, $G_{i',j'}$, and $\hat{p}$. To this end we start by first estimating $Var(\hat{p})$ and $E[(G_{i^{'},j^{'}}-2 p)( \hat{p}-p)]$. Relationship (1) and (2) from main manuscript [also in Devlin et al.(*1*)], can be re-written as: ${Var(G}_{i,j})={Var(G'}_{i,j})=2p\left( 1-p \right)(1+F_{i})$ and $Cov\left( G_{i,j},G_{i,j^{'}} \right)=Cov\left( {G'}_{i,j},G_{i,j^{'}} \right)=Cov\left( G_{i,j},{G'}_{i,j^{'}} \right)=4 p(1-p)F_{i}$ and $Cov\left( G_{i^{'},j},G_{i,j^{'}} \right)=0$ for $i^{'}\neq i$ .

With these relationships $Var\left( \hat{p} \right)=Var\left( \frac{\omega}{2 n} \sum_{i=1}^{m} \sum_{j=1}^{n_{i}} G_{i,j}+\frac{\left( 1-\omega\right)}{2 n^{'}} \sum_{i=1}^{m} \sum_{j=1}^{{n^{'}}_{i}} {G^{'}}_{i,j} \right)$ becomes $Var\left( \hat{p} \right)=2p\left( 1-p \right)\{\frac{\omega^{2}}{4 n^{2}}[n(1+F_{i})+2 \sum_{i=1}^{m} n_{i}\left( n_{i}-1 \right)F_{i}] +\frac{4\omega\left( 1-\omega\right)}{4 n n^{'}}\sum_{i=1}^{m} n_{i}{n^{'}}_{i}F_{i}+\frac{\left( 1-\omega\right)^{2}}{4 {n^{'}}^{2}}[n^{'}(1+F_{i})+2 \sum_{i=1}^{m} {n^{'}}_{i}\left( {n^{'}}_{i}-1 \right)F_{i}]\}$. Similarly, $E[(G_{i^{'},j^{'}}-2 p)( \hat{p}-p)]=E[(G_{i^{'},j^{'}}-2 p)[\frac{\omega}{2 n} \sum_{i=1}^{m} \sum_{j=1}^{n_{i}} {(G}_{i,j}-2 p)+\frac{\left( 1-\omega\right)}{2 n^{'}} \sum_{i=1}^{m} \sum_{j=1}^{{n^{'}}_{i}} {{(G}^{'}}_{i,j}-2 p)]\}$ simplifies to

$E[(G_{i^{'},j^{'}}-2p)( \hat{p}-p)]=2p\left( 1-p \right)\{\frac{\omega}{2 n}2*n_{i^{'}}F_{i^{'}}+2{\frac{\left( 1-\omega\right)}{2 n^{'}}n^{'}}_{i^{'}}F_{i^{'}}\}$. Thus, the correlation of interest becomes:

$Cor\left( G_{i',j'} \hat{p} \right)=\frac{\frac{\omega}{n}n_{i^{'}}F_{i^{'}}+\frac{\left( 1-\omega\right)}{n^{'}}{{n'}_{i^{'}}F}_{i^{'}}}{\sqrt{(1+F_{i})(\frac{\omega^{2}}{4 n^{2}}\left[ n\left( 1+F_{i} \right)+2 \sum_{i=1}^{m} n_{i}\left( n_{i}-1 \right)F_{i} \right] +\frac{4\omega\left( 1-\omega\right)}{4 n n^{'}}\sum_{i=1}^{m} n_{i}{n^{'}}_{i}F_{i}+\frac{\left( 1-\omega\right)^{2}}{4 {n^{'}}^{2}}\left[ n^{'}\left( 1+F_{i} \right)+2 \sum_{i=1}^{m} {n^{'}}_{i}\left( {n^{'}}_{i}-1 \right)F_{i} \right])}}$.

Further manipulations, reduces the correlation to:

$Cor\left( G_{i',j'} \hat{p} \right)=\frac{2 n_{i^{'}}F_{i^{'}}+2 {\frac{\left( 1-\omega\right)n}{\omega n'}n}_{i^{'}}F_{i^{'}}}{\sqrt{\left( 1+F_{i} \right)[n(1+F_{i})+2\sum_{i=1}^{m} n_{i}\left( n_{i}-1 \right)F_{i}] +\frac{4\left( 1-\omega\right)n}{\omega n^{'}}\sum_{i=1}^{m} n_{i}{n^{'}}_{i}F_{i}+\frac{\left( 1-\omega\right)^{2} n^{2}}{\omega^{2}{n^{'}}^{2}}[n^{'}(1+F_{i})+2 \sum_{i=1}^{m} {n^{'}}_{i}\left( {n^{'}}_{i}-1 \right)F_{i}]}}$.

If we assume the same $F_{st}$ for all populations and an equal number of cases and controls in each subpopulations, i.e. $F_{i}=F$ and $n_{i}={n'}_{i}=\frac{n}{m}$, for large numbers the formula is approximated by: $Cor\left( G_{i',j'} \hat{p} \right)\cong\frac{2 \left[ 1+\frac{\left( 1-\omega\right)}{\omega} \right]\frac{n}{m}F}{\sqrt{(1+F)(\left[ 1+\frac{\left( 1-\omega\right)^{2}}{\omega^{2}} \right]n +\left[ 1+\frac{2 \left( 1-\omega\right)}{\omega}+{\frac{\left( 1-\omega\right)}{\omega}}^{2} \right]\frac{{2 n}^{2}}{m}F)}}$

**1.2 Unknown** $p$

To compute $Cor\left( G_{i',j'} \hat{p} \right)$ one needs the value for the mixed ethnicity MAF, $p$. However, this is rarely known exactly. In practice, when compared to known $p$, the need to estimate such quantity decreases the strength of the available information. Thus, intuitively $Cor\left( G_{i',j'} \hat{p} \right)$ can be viewed as an upper bound of the effect size. As many of our conservative upper bound choices, this will assist in providing an upper bound for the power to detect SBCC signal.

**1.3 Correlation between a case genotype and RAF of all cases**

****When reporting RAF for all cases ($\omega=1$), which might be perceived as the worst-case scenario privacy-wise, the expected correlation between a case genotype and the RAFs from the above equation becomes $\rho\left( F \right)=Cor\left( G_{i',j'} \hat{p} \right)\cong\frac{1+2 \frac{n}{m}F}{\sqrt{(1+F)(n +\frac{{2 n}^{2}}{m}F)}}$. (A)

Figure A. Empirical validation of correlation $\rho\left( F \right)$

This relationship was successfully verified empirically (Figure A) using a beta-binomial simulation as described in Devlin et al. (*2*), for various values (Table A) of the parameters. To obtain the exact correlation between$Cor\left( G_{i',j'} \hat{p} \right)$, e.g. $p$ to be practically the same for all markers, $p$ was maintained within tight limits ($0.001$) around the desired value of, i.e. $\{0.05; 0.15; 0.25; 0.35; 0.45\}$ in Table A. The successful verification of equation (1), is underscored by the exceptionally high coefficient of determination, R^2^=99.99%, between the predicted and observed genotype-AF $\left( G_{i',j'} \hat{p} \right)$correlation coefficients.

A first order Taylor approximation for relationship (1) around $F=0$, yields (for $n>>m$ and moderate $F$):

$\rho\left( F \right)\cong\frac{1}{\sqrt{n}} [1+\frac{n-\frac{m}{2}}{m}F$] $\cong\frac{1}{\sqrt{n}} \left[ 1+\frac{n}{m} F \right]=\rho\left( 0 \right)+\frac{\sqrt{n}}{m} F>\rho\left( 0 \right)=\frac{1}{\sqrt{n}}$ (B).

It follows that, if $\hat{F}$ be the in-study within subject correlation of alleles, $\hat{\rho\left( F \right)}\cong\hat{\rho\left( 0 \right)}+\frac{\sqrt{n}}{m} \hat{F}$ (C). Inferences regarding a subject belonging to a cohort should be, consequently, made based on $\hat{\rho\left( 0 \right)}$, i.e. comparing it to $\frac{1}{\sqrt{n}}$. Thus, for large intra(inter)-continental cohorts the theoretical expected correlation due to case inclusion might be heavily influenced by $F$, for which the sample estimated is not reported by studies.

Table A. Values of parameters used for obtaining Figure A.

| Parameter | Values |
| --- | --- |
| N [thousands] | {1, 2, 5} |
| Fst | {0.001, 0.003, 0.007, 0.01} |
| m | {2, 4, 10, 20, 50} |
| p [%] | {5, 15, 25, 35, 45} |

**1.4 Variance of** $\hat{\rho\left( 0 \right)}$ **estimators**

The ~$\frac{\sqrt{n}}{m} F$ upward bias can be eliminated from the more exact relation (Equation A), albeit only by retorting to skill intensive techniques based on study description to estimate the unreported$\hat{F}$. Let $o$ be the equivalent number of independent markers in the genome scans and $\hat{F}$ be the in-study within subject correlation of alleles. As it can be interpreted as a correlation estimate obtained from $o$ observations, $\rho\left( \hat{F} \right)$ has then variance $Var[\rho\left( \hat{F} \right) ]=\frac{1}{o-3}$. However, because $\hat{F}$ is practically never reported, it needs to be estimated, e.g. from the sample or a relevant (*and close-to-perfectly matched*) reference panel of size $n^{''}$. Let $k=\frac{n}{n''}$ (which is typically >> 10 for large meta-analyses). Let $\tilde{F}$, be the estimate from such a matched panel.

For testing $\rho\left( 0 \right)=\frac{1}{\sqrt{n}}$ vs. $\rho\left( 0 \right)=0$, we do not know $\rho\left( 0 \right)$ and need to estimate it from $\hat{F}$ and $\hat{\rho}\left( \hat{F} \right)$. Because from (2) $\hat{\rho\left( 0 \right)}$ $\cong\hat{\rho\left( F \right)}-\frac{\sqrt{n}}{m} \tilde{F}$, then $Var\left[ \hat{\rho\left( 0 \right)} \right]= Var\left[ \hat{\rho\left( F \right)} \right]+Var\left[ \frac{\sqrt{n}}{m} \tilde{F} \right]=\frac{1}{o}+\frac{n}{m^{2}}\frac{1}{n''}=\frac{1}{o}+\frac{k}{m^{2}}$ (D).

Reference List

1. B. Devlin, K. Roeder, L. Wasserman, *Theor. Popul. Biol.* **60**, 155 (2001).

2. B. Devlin, S. A. Bacanu, K. Roeder, *Nat. Genet.* **36**, 1129 (2004).
